# Supplementary material for: Effect of Cyclin-Dependent Kinase 4/6 Inhibitors on Circulating Cells in Patients with Metastatic Breast Cancer
Source: Cells. 2024 Aug 21;13(16):1391. doi: 10.3390/cells13161391 (PMC11487375; doi:10.3390/cells13161391)
Supplement: Supplementary file 1 [file cells-13-01391-s001.zip › cells-3140560-supplementary.pdf]

Supplementary Annex 1: Inclusion and Exclusion Criteria for Sub-Analysis of CDK4/6 Inhibitors in ER+/HER2- Metastatic Breast Cancer

Inclusion and exclusion criteria for the ONCODYNAMICS BioBanking (ODB) Study

The ONCODYNAMICS BioBanking (ODB) project is a comprehensive initiative designed to support precision medicine in cancer by evaluating tumor clonal evolution, host immune response, and circulating biomarkers. The inclusion criteria for patients enrolled in the ODB project are as follows:

- Patients must provide written informed consent to participate in the study and allow the use of their clinical data and biological samples for research purposes.
- Patients must be 18 years of age or older and have an Eastern Cooperative Oncology Group (ECOG) performance status (PS) of 0-2 at the time of enrollment.
- Patients must have a confirmed diagnosis of advanced/metastatic cancer, specifically one of the following types: breast cancer, colorectal cancer, malignant melanoma, and prostate cancer.
- Patients must be undergoing active anti-cancer systemic treatment as per the Oncology Department guidelines. This includes but is not limited to chemotherapy, targeted therapy, immunotherapy, and endocrine therapy.
- Patients must agree to adhere to the follow-up schedule as outlined in the study protocol, which includes regular clinical evaluations and sample collections. Patients must agree to provide blood samples at baseline and at specified intervals throughout the study.

Inclusion and Exclusion Criteria for this subgroup analysis

<u>Inclusion Criteria:</u>

1. Female patients.
2. Confirmed diagnosis of ER+/HER2- advanced/metastatic breast cancer
3. Patients must be treated with CDK4/6 inhibitors (CDK4/6i) in combination with endocrine therapy (ET) in either the first- or second line for metastatic setting.
4. Patients must have comprehensive clinical data available, including demographic, clinical, and pathological information, as well as follow-up data.
5. Patients must have a minimum of months of follow-up data to allow for categorization into responder or non-responder groups.

<u>Exclusion Criteria:</u>

1. HER2-Positive or Triple-Negative breast cancer.
2. Use of CDK4/6 inhibitors in later lines or in monotherapy.
3. Other active malignancies that could impact survival outcomes were excluded, except for those with adequately treated basal or squamous cell carcinoma or carcinoma in situ of the cervix.

Supplementary S1: FACS gating strategy

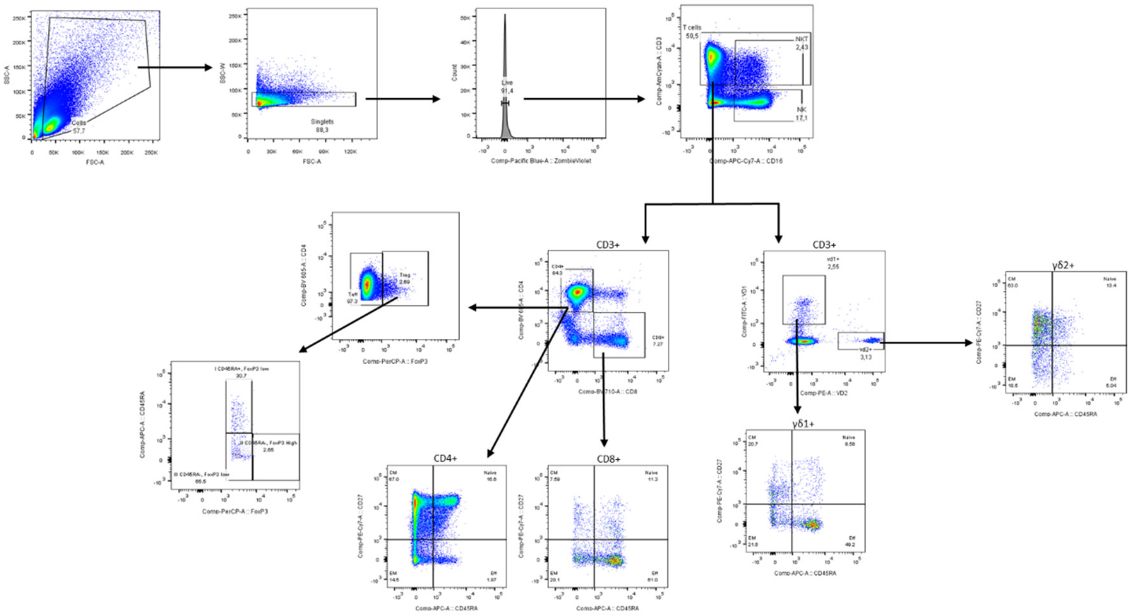

Supplementary data Figure S1 A) FACS gating strategy – Mix A

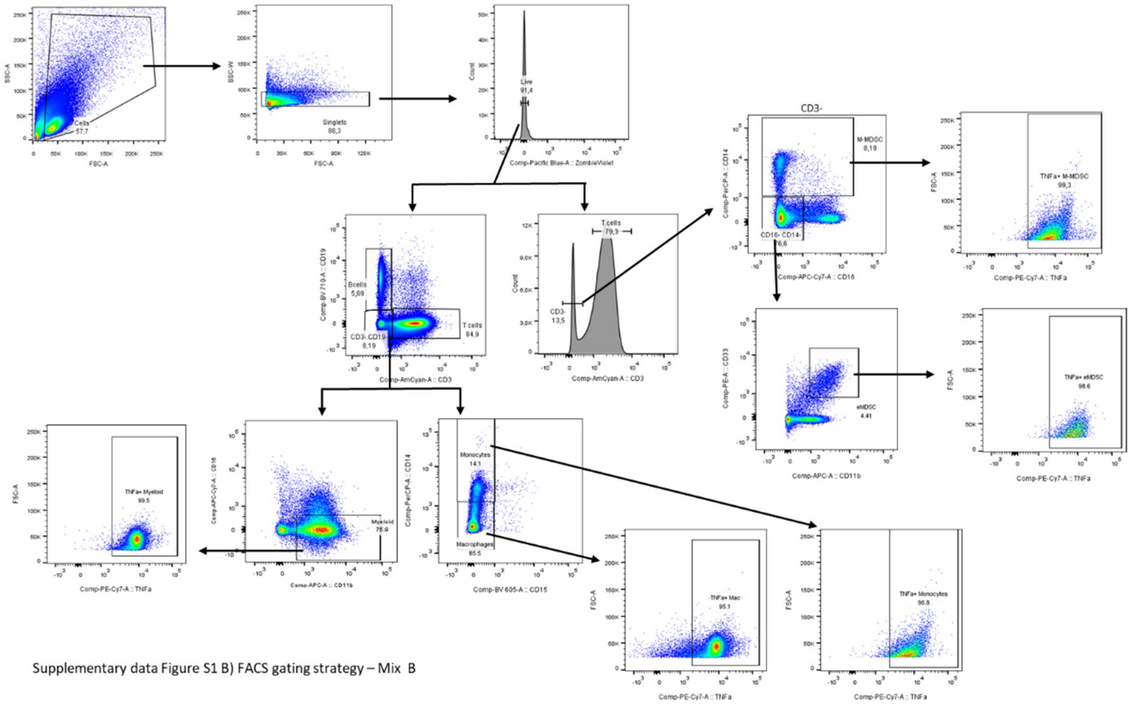

Supplementary data Figure S1 B) FACS gating strategy – Mix B

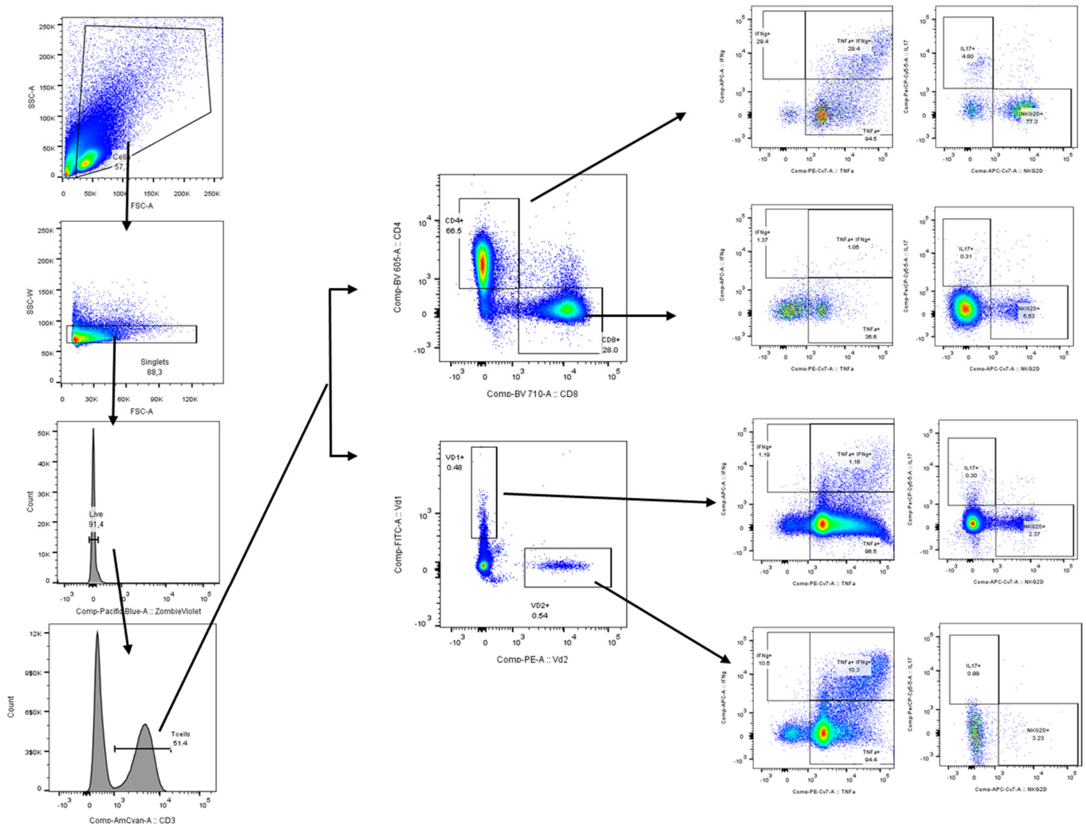

Supplementary data Figure S1 C) FACS gating strategy – Mix C

Supplementary Table S1: anti-human fluorescently labeled mAbs used in the FACS staining

Antibody	**Fluorophore**	**Clone**	**Mix**
Anti-human Foxp3	PerCP-Cyanine5.5	PCH101	A
Anti-CD27	PE-Cyanine7	LG.7F9	A
Anti-human CD314 (NKG2D)	APC/Cy7	1D11	C
Anti-human CD11b	APC	ICRF44	B
Anti-human IL-17A	PerCP/Cy5.5	BL168	C
Anti-human IFN-γ	APC	4S.B3	C
Anti-human TNF-α	PE/Cy7	Mab11	C
Anti-human CD15 (SSEA-1)	Brilliant Violet 605	W6D3	B
Anti-human CD16	APC/Cy7	3G8	A, C
Anti-human CD3	Brilliant Violet 510	OKT3	A, B, C
Anti-human TCR Vδ2	PE	B6	A,C
Anti-human CD4	Brilliant Violet 605	OKT4	A, B, C
Anti-human CD8a	Brilliant Violet 711	RPA-T8	A, B, C
Anti-human CD33	PE	P67.6	B
Anti-human CD19	Brilliant Violet 711	HIB19	B
Anti-human CD45RA	APC	HI100	A
Anti-human CD14	PerCP/Cy5.5	M5E2	B
Zombie Violet™ Fixable Viability Kit	Pacific blue	-	A, B, C
TCR V delta 1 Monoclonal Antibody	FITC	TS8.2	A, C

Supplementary Table S2: significance of variables for the baseline characteristics

Variable	**p-value**
TotalT	0.7652
NK	0.2693
NKT	0.5307
CD4	0.6347
CD4CM	0.8696
CD4Eff	0.3135
CD4EM	0.8228
CD4N	0.6543
Teff	0.2133
Treg	0.9234
TregI	0.2324
TregII	0.5307
TregIII	0.0916
CD4Mem	0.8813
CD8	0.6118
CD8CM	0.6790
CD8Eff	0.5706
CD8EM	0.6223
CD8N	0.7653
CD8Mem	0.3045
VD1	0.5017
VD1CM	0.3101
VD1Eff	0.7201
VD1EM	0.4202
VD1N	0.3394
VD1Mem	0.9524
VD2	0.7826
VD2CM	0.1785
VD2Eff	0.0394
VD2EM	0.1006
VD2N	0.7174
VD2Mem	0.2825
CD4IFNg	0.9458
CD4NKG2D	0.9708
CD4IL17	0.1968
CD4TNFa	0.9250
CD4TNFaIFNg	0.7857
CD8IFNg	0.2268
CD8IL17	0.1102
CD8NKG2D	0.2355
CD8TNFa	0.7345
CD8TNFaIFNg	0.2593

VD1IFNg	0.6171
VD1IL17	0.1933
VD1NKG2D	0.3045
VD1TNFa	0.6378
VD1TNFaIFNg	0.7290
VD2IFNg	0.4189
VD2IL17	0.7263
VD2NKG2D	0.4690
VD2TNFa	0.8836
VD2TNFaIFNg	0.1310
B	0.6979
eMDSC	0.5897
MDSC	0.5697
Myel	0.4589
MyelTNFa	0.4711
VD2Eff.VD2CM	0.2215
CD8Eff.CD8CM	0.8421
CD8Eff.CD8Mem	0.1823
Teff.Treg	0.8307
CD4.CD8	0.5916
CD4eff.CD8eff	0.5286
CD8.Treg	0.8728
TregIII.TregII	0.7882
Hb	0.7954
MCV	0.1737
Leuc	0.6619
Neut	0.9483
Eosi	0.2633
Baso	0.0054
Linf	0.2436
NLR	0.3506
Mono	0.8265
Plaq	0.5598
sIFNg	0.7265
LDH	0.0860
CEA	0.0077
CA15.3	0.3393

Supplementary Table S3: Correlation of immune cell populations with the presence of CTCs

Variable	r	p-value
TotalT	-0.1989	0.3748
NK	0.0656	0.7719
NKT	-0.2299	0.3033
CD4	0.3252	0.1397
CD4CM	0.2892	0.1918
CD4Eff	0.0605	0.7891
CD4EM	-0.2303	0.3026
CD4N	-0.0233	0.9179
Teff	0.1439	0.5229
Treg	0.0749	0.7405
TregI	-0.2219	0.3210
TregII	0.1860	0.4072
TregIII	-0.0573	0.8000
CD4Mem	0.0343	0.8795
CD8	-0.1559	0.4885
CD8CM	0.6827	0.0189
CD8Eff	-0.6923	0.0004
CD8EM	-0.3019	0.1721
CD8N	0.3403	0.1212
CD8Mem	0.2732	0.2187
VD1	-0.4664	0.0287
VD1CM	0.4960	0.0005
VD1Eff	-0.4605	0.0310
VD1EM	-0.2314	0.3002
VD1N	0.4392	0.0408
VD1Mem	0.1693	0.4512
VD2	-0.0771	0.7331
VD2CM	0.1831	0.4148
VD2Eff	-0.1034	0.6469
VD2EM	-0.1845	0.4112
VD2N	0.1647	0.4639
VD2Mem	0.0470	0.8354
CD4IFNg	NA	NA
CD4NKG2D	-0.3118	0.1577
CD4IL17	0.0919	0.6842
CD4TNFa	NA	NA
CD4TNFaIFNg	0.4734	0.0260
CD8IFNg	0.3645	0.0954
CD8IL17	0.0232	0.9185
CD8NKG2D	0.2861	0.1967
CD8TNFa	-0.3243	0.1409
CD8TNFaIFNg	0.1958	0.3826

VD1IFNg	0.3506	0.1097
VD1IL17	-0.0308	0.8918
VD1NKG2D	-0.4727	0.0263
VD1TNFa	-0.3979	0.0667
VD1TNFaIFNg	0.2804	0.2062
VD2IFNg	0.0951	0.6736
VD2IL17	-0.2489	0.2641
VD2NKG2D	-0.3248	0.1403
VD2TNFa	-0.2901	0.1904
VD2TNFaIFNg	0.0021	0.9925
B	0.0108	0.9620
eMDSC	-0.3065	0.1653
MDSC	-0.0848	0.7074
Myel	-0.1799	0.4231
MyelTNFa	-0.2490	0.2638
VD2Eff.VD2CM	-0.0491	0.8283
CD8Eff.CD8CM	-0.4788	0.0242
CD8Eff.CD8Mem	-0.4380	0.0415
Teff.Treg	-0.1494	0.5069
CD4.CD8	0.2728	0.2194
CD4eff.CD8eff	0.1546	0.4921
CD8.Treg	-0.1994	0.3737
TregIII.TregII	-0.3704	0.0897
Hb	0.0230	0.9191
MCV	0.0514	0.8203
Leuc	0.2503	0.2613
Neut	0.2808	0.2055
Eosi	0.2062	0.3572
Baso	-0.1538	0.4943
Linf	-0.0275	0.9034
NLR	0.2519	0.2581
Mono	-0.1952	0.3839
Plaq	0.2467	0.2684
sIFNg	-0.4319	0.0447
LDH	-0.2441	0.2736
CEA	-0.4030	0.0630
CA15.3	-0.3273	0.1371

Supplementary Table S4: Significance of the impact of CDK4/6i treatment on immune populations

Variable	p-value
TotalT	0.2226
NK	0.2055
NKT	0.6010
CD4	0.1045
CD4CM	0.0078
CD4Eff	0.2514
CD4EM	0.7090
CD4N	0.4452
Teff	0.6556
Treg	0.6102
TregI	0.0738
TregII	0.3880
TregIII	0.8697
CD4Mem	0.2802
CD8	0.1895
CD8CM	0.6333
CD8Eff	0.5202
CD8EM	0.4100
CD8N	0.7998
CD8Mem	0.6650
VD1	0.2726
VD1CM	0.4452
VD1Eff	0.7745
VD1EM	0.9854
VD1N	0.7502
VD1Mem	0.8462
VD2	0.4140
VD2CM	0.1375
VD2Eff	0.2877
VD2EM	0.3038
VD2N	0.4091
VD2Mem	0.4060
CD4IFNg	0.7596
CD4IL17	0.3857
CD4NKG2D	0.1650
CD4TNFa	0.1678
CD4TNFaIFNg	1.0000
CD8IFNg	0.4212
CD8IL17	0.8438
CD8NKG2D	0.6095
CD8TNFa	0.1126

CD8TNFaIFNg	0.4263
VD1IFNg	0.5693
VD1IL17	0.1563
VD1NKG2D	0.8124
VD1TNFa	0.0826
VD1TNFaIFNg	0.8203
VD2IFNg	0.6772
VD2IL17	0.6698
VD2NKG2D	0.0122
VD2TNFa	0.1126
VD2TNFaIFNg	0.6772
B	0.9643
eMDSC	0.0001
MDSC	0.6788
Myel	0.6742
MyelTNFa	0.1140
VD2Eff.VD2CM	1.0000
CD8Eff.CD8CM	0.7998
CD8Eff.CD8Mem	0.7454
Teff.Treg	0.5854
CD4.CD8	0.0605
CD4eff.CD8eff	0.3442
CD8.Treg	0.7024
TregIII.TregII	0.9746
Hb	0.0003
MCV	0.0008
Leuc	0.0000
Neut	0.0001
Eosi	0.0043
Baso	0.3425
Linf	0.1769
NLR	0.0012
Mono	0.0130
Plaq	0.5882
sIFNg	0.9622
LDH	NA
CEA	NA
CA15.3	NA

Supplementary Table S5: Variation in immune cell subsets according to response to CDK4/6i

	**t2: Resp Vs NResp**	**delta t2-baseline**
Variable	p-value	p-value
TotalT	0.5528	0.2226
NK	0.8551	0.2055
NKT	0.3374	0.6010
CD4	0.7493	0.1045
CD4CM	0.9272	0.0078
CD4Eff	0.5528	0.2514
CD4EM	0.6979	0.7090
CD4N	0.8300	0.4452
Teff	0.6479	0.6556
Treg	0.8978	0.6102
TregI	0.9238	0.0738
TregII	0.5234	0.3880
TregIII	0.6347	0.8697
CD4Mem	0.9656	0.2802
CD8	0.5227	0.1895
CD8CM	0.8801	0.6333
CD8Eff	0.8551	0.5202
CD8EM	0.5726	0.4100
CD8N	0.7634	0.7998
CD8Mem	0.5726	0.6650
VD1	0.7493	0.2726
VD1CM	0.9656	0.4452
VD1Eff	0.8571	0.7745
VD1EM	0.3579	0.9854
VD1N	1.0000	0.7502
VD1Mem	0.8300	0.8462
VD2	0.7870	0.4140
VD2CM	0.5857	0.1375
VD2Eff	0.5338	0.2877
VD2EM	0.9199	0.3038
VD2N	0.9211	0.4091
VD2Mem	0.4636	0.4060
CD4IFNg	1.0000	1.0000
CD4IL17	0.2143	1.0000
CD4NKG2D	0.6026	0.1650
CD4TNFa	0.4000	0.1678
CD4TNFaIFNg	0.8037	1.0000
CD8IL17	1.0000	0.8438
CD8IFNg	0.4421	1.0000
CD8NKG2D	0.4822	0.6095
CD8TNFa	0.2549	0.1126

CD8TNFaIFNg	0.4421	0.4263
VD1IFNg	0.3323	1.0000
VD1IL17	1.0000	0.1563
VD1NKG2D	1.0000	0.8124
VD1TNFa	0.2381	0.0826
VD1TNFaIFNg	0.3810	0.8203
VD2IFNg	0.3877	1.0000
VD2IL17	0.1324	0.6698
VD2NKG2D	0.3750	0.0122
VD2TNFa	0.5333	0.1126
VD2TNFaIFNg	0.4211	0.6772
B	0.3094	0.9643
eMDSC	0.0087	0.0000
MDSC	0.6737	0.6788
Myel	0.2857	0.6742
MyelTNFa	0.2857	0.1140
VD2Eff.VD2CM	0.3106	1.0000
CD8Eff.CD8CM	0.6979	0.7998
CD8Eff.CD8Mem	0.6979	0.7454
Teff.Treg	0.8978	0.5854
CD4.CD8	0.6979	0.0605
CD4eff.CD8eff	0.8106	1.0000
CD8.Treg	0.6347	0.7024
TregIII.TregII	0.3078	0.9746
Hb	0.3920	0.0003
MCV	1.0000	0.0008
Leuc	0.0466	0.0000
Neut	0.2617	0.0001
Eosi	0.7223	0.0043
Baso	0.0142	0.3425
Linf	0.1314	0.1769
NLR	0.6692	0.0759
Mono	0.0968	0.0130
Plaq	0.8872	0.5882
sIFNg	0.7505	0.9622
LDH	NA	NA
CEA	NA	NA
CA15.3	NA	NA
